# Determining Acceptance of e-Mental Health Interventions in Digital Psychodiabetology Using a Quantitative Web-Based Survey: Cross-sectional Study

**DOI:** 10.2196/27436

**Published:** 2021-07-30

**Authors:** Mirjam Damerau, Martin Teufel, Venja Musche, Hannah Dinse, Adam Schweda, Jil Beckord, Jasmin Steinbach, Kira Schmidt, Eva-Maria Skoda, Alexander Bäuerle

**Affiliations:** 1 Clinic for Psychosomatic Medicine and Psychotherapy LVR-University Hospital Essen, University of Duisburg Essen Essen Germany

**Keywords:** e-mental health, acceptance, UTAUT, mental health, diabetes, e-mental health intervention, psychodiabetology

## Abstract

**Background:**

Diabetes is a very common chronic disease that exerts massive physiological and psychological burdens on patients. The digitalization of mental health care has generated effective e-mental health approaches, which offer an indubitable practical value for patient treatment. However, before implementing and optimizing e-mental health tools, their acceptance and underlying barriers and resources should be first determined for developing and establishing effective patient-oriented interventions.

**Objective:**

This study aims to assess the acceptance of e-mental health interventions among patients with diabetes and explore its underlying barriers and resources.

**Methods:**

A cross-sectional study was conducted in Germany from April 9, 2020, to June 15, 2020, through a web-based survey for which patients were recruited via web-based diabetes channels. The eligibility requirements were adult age (18 years or older), a good command of the German language, internet access, and a diagnosis of diabetes. Acceptance was measured using a modified questionnaire, which was based on the well-established Unified Theory of Acceptance and Use of Technology (UTAUT) and assessed health-related internet use, acceptance of e-mental health interventions, and its barriers and resources. Mental health was measured using validated and established instruments, namely the Generalized Anxiety Disorder Scale-7, Patient Health Questionnaire-2, and Distress Thermometer. In addition, sociodemographic and medical data regarding diabetes were collected.

**Results:**

Of the 340 participants who started the survey, 261 (76.8%) completed it and the final sample comprised 258 participants with complete data sets. The acceptance of e-mental health interventions in patients with diabetes was overall moderate (mean 3.02, SD 1.14). Gender and having a mental disorder had a significant influence on acceptance (*P*<.001). In an extended UTAUT regression model (UTAUT predictors plus sociodemographics and mental health variables), distress (β=.11; *P*=.03) as well as the UTAUT predictors *performance expectancy* (β=.50; *P*<.001), *effort expectancy* (β=.15; *P*=.001), and *social influence* (β=.28; *P*<.001) significantly predicted acceptance. The comparison between an extended UTAUT regression model (13 predictors) and the UTAUT-only regression model (*performance expectancy*, *effort expectancy*, *social influence*) revealed no significant difference in explained variance (*F*_10,244_=1.567; *P=*.12).

**Conclusions:**

This study supports the viability of the UTAUT model and its predictors in assessing the acceptance of e-mental health interventions among patients with diabetes. Three UTAUT predictors reached a notable amount of explained variance of 75% in the acceptance, indicating that it is a very useful and efficient method for measuring e-mental health intervention acceptance in patients with diabetes. Owing to the close link between acceptance and use, acceptance-facilitating interventions focusing on these three UTAUT predictors should be fostered to bring forward the highly needed establishment of effective e-mental health interventions in psychodiabetology.

## Introduction

### Background

Diabetes is a common chronic disease that exerts a heavy physiological and psychological burden on patients. In 2017, approximately 451 million adult patients were affected worldwide, and the number is steadily increasing [1]. As diabetes is considered to be “one of the most psychologically demanding of chronic medical illnesses” [2], the integration of psychological care in the management of diabetes is crucial [3,4].

Living with diabetes means facing day-to-day challenges and complications resulting in considerable emotional distress [5], which can lead to a higher risk of psychological disorders. Indeed, patients with diabetes show disproportionately higher rates of psychological disorders than those without diabetes [5], including depression and anxiety [6,7]. Considering psychological comorbidity, diabetes mellitus is often accompanied by depression (18.8%-24%) [8-10]. They occur twice as frequently together as predicted by chance alone and worsen each other because of underlying biological and behavioral mechanisms such as diabetes-related symptoms and sleep disorders [11,12]. A meta-analysis including more than 50,000 participants confirmed the major role of depression as a comorbidity of diabetes, and found that participants with type 2 diabetes had significantly higher depression rates than those without diabetes (17.6% vs 9.8%; odds ratio 1.6, 95% CI 1.2-2.0) [13]. Regarding the prevalence of anxiety, a systematic review found that 14% of patients with diabetes who participated in clinical studies with generalized anxiety disorder and 27% with subsyndromal anxiety disorder [14]. Considering that 40% of their participants expressed the elevated symptoms of anxiety (N=1283 in 7 studies), anxiety turned out to be a major mental health threat to people with diabetes.

The psychological vulnerability of people with diabetes manifests itself in the heightened risks of psychological disorders; for example, the risk of developing depression is 24% higher in patients with type 2 diabetes than in individuals without diabetes [15]. However, diabetes and mental health appear to exert a bidirectional effect on each other because poor mental health is associated with an increased risk of developing type 2 diabetes [16]. Psychological support and self-empowerment are crucial, as diabetes impairs the psychological well-being and quality of life [17]. Although diabetes-related self-management education programs can reduce diabetes-related as well as emotional distress [5,18], help foster self-efficacy [18] and have proven their effectiveness [19] and cost-effectiveness [20] as demonstrated by web-based structured education programs, they are still not implemented to a sufficient extent in routine diabetes care [21]. Therefore, improving diabetes-related knowledge and self-care practices should be a major goal in diabetes management to reduce the risks of developing and chronifying psychological disorders [5,22].

The digitalization of mental health care has generated effective e-mental health approaches, which have an indubitable practical value for patient treatment [23-26]. The effects of e-mental health interventions for some mental disorders are comparable with those of a traditional face-to-face therapy [26]. Nonetheless, web-based interventions offer several advantages that cannot be provided by offline interventions, for example, offering an anonymous, low-threshold, cost-effective, time- and location-flexible alternative [27]. However, limited accessibility, negative treatment expectancies, and concerns about anonymity create serious challenges in the implementation of e-mental health approaches. To date, existing e-mental health interventions have mainly focused on psychosomatic inpatients and provide information and interactive tasks based on cognitive behavioral, psychodynamic, or acceptance and commitment therapy [28]. Unfortunately, clinical e-mental health interventions are still scarce and are not well known among patients in Germany [29]. Although patients with diabetes can make use of several apps and at least some (web-based) diabetes education programs that help in disease management [30,31], only a few web-based e-mental health interventions exist. A recent systematic review from 2021 found 9 studies offering digital interventions for psychological comorbidities in patients with diabetes [32]. Two of these studies were conducted in Germany [33,34] and both offered the guided self-help web-based intervention GET.ON Mood Enhancer Diabetes for depression in people with diabetes. Furthermore, 7 out of these 9 studies found the offered e-mental health intervention to be effective in terms of improvements in depressive symptoms [33-39] and 4 found the intervention effective with respect to diabetes (specific emotional) distress [34,37-39]. Given the current situation concerning the COVID-19 pandemic, the need for innovative and easily accessible approaches in psychological care is evident and has also increased. There is a scientific consensus that depression, anxiety, sleeping problems, and stress have increased over the course of the ongoing pandemic [40-45]. With the knowledge of the vulnerability of people with diabetes and in light of the pandemic and its mental health implications, we conducted a study to investigate the acceptance of e-mental health interventions in patients with diabetes.

As previous research suggests that patients with diabetes having depression symptoms might have a low motivation to participate in a screening program or psychological care [46], the acceptance of (future) interventions is a major variable that research should be considered. Thus, before implementing and optimizing e-mental health tools, their acceptance and underlying barriers and resources should first be examined and understood. In terms of usefulness, even the best intervention can only be beneficial to those patients using it. Therefore, the determinants of acceptance and uptake of e(-mental) health interventions need to be further analyzed. The research on eHealth acceptance has harnessed the Unified Theory of Acceptance and Use of Technology (UTAUT) for assessing the predictors of behavioral intention and acceptance [28,47-51]. The UTAUT model contributed to the analysis of factors that influence the acceptance of e-mental health interventions in patients with diabetes [52]. This model and its e-mental health specific extensions [28,52] enabled researchers to tailor interventions properly to specific patient groups, because acceptance-influencing determinants were known from analysis with the UTAUT. Unfortunately, barely any validation of the UTAUT has been investigated in e-mental health programs for patients with diabetes. However, there is some evidence in disease management programs. For example, a survey of 116 patients with diabetes used the UTAUT model to identify the factors that influence the acceptance of telemedicine services. Researchers have found that performance expectancy (PE), effort expectancy (EE), and social influence (SI) are significant factors that contribute to the acceptance of telemedicine services for diabetes management. In addition, gender and age were identified as moderators between PE and acceptance as predicted by the UTAUT model [53].

The UTAUT postulates four core predictors: *PE*, *EE*, *SI*, and *facilitating conditions* (*FC*) [54]. Acceptance itself is operationalized as *behavioral intention*, which is predicted by the first three core predictors, whereas actual use is predicted by *behavioral intention* and *FC*. *PE* describes the degree to which an individual believes that using a system will be helpful. *EE* is defined as the degree of ease associated with the use of a system. *SI* describes the degree to which an individual perceives that important other, such as family or friends, would approve of the use of the system. *FC* are defined as the degree to which an individual believes that an organizational and technical infrastructure exists to support the use of a system. By applying UTAUT as a method of measuring acceptance, the notable values of explained variance (70%) can be achieved [54]. To prove external validity and generalizability, UTAUT needs to be explored in different target groups. Given the massive physiological and psychological burdens caused by diabetes, this study focused on investigating the acceptance of e-mental health interventions in a sample of patients with diabetes.

### Objectives

This study aims to determine the acceptance of e-mental health interventions among patients with diabetes and explore the underlying factors influencing patients’ intentions to use such interventions. The acceptance of e-mental health interventions is associated with sociodemographic characteristics such as gender, age, and education [28,55] and mental health, such as anxiety and current or past mental disorders [48,55]. Therefore, we extended the UTAUT model and added sociodemographic, medical, and validated mental health variables as the direct predictors of acceptance. In addition, this study aims to examine the viability of the UTAUT model with its three predictors of acceptance (*PE*, *EE*, and *SI*) in assessing patients with diabetes’ acceptance of e-mental health interventions and to investigate whether this extended UTAUT model proves to be superior and more effective. Furthermore, an additional goal is to examine whether age and gender modulate the relationship of *PE*, *EE*, *SI* and acceptance, respectively, as postulated by the UTAUT [54]. Thus, the following research questions are addressed:

1. To what extent do patients with diabetes accept e-mental health interventions and does acceptance differ significantly regarding sociodemographic or medical characteristics of the participants?
2. Is the proposed extended UTAUT model suitable to predict the acceptance of e-mental interventions in patients with diabetes and which factors are significant predictors?
3. Is the proposed extended UTAUT model superior to the UTAUT model and do age and gender modulate the relationship between each UTAUT predictor and acceptance?

Answers to the research questions above might contribute to the process of implementing and improving e-mental health interventions. Especially during the ongoing COVID-19 pandemic, these results may be beneficial to individuals with mental health problems or those at a higher risk of developing psychological disorders.

## Methods

### Study Design and Participants

A cross-sectional study was conducted to assess the acceptance of e-mental health interventions and its underlying predictors in patients with diabetes. The Checklist for Reporting Results of Internet E-Surveys was used to report the methods and results of our web-based open survey [56]. From April 9 to June 15, 2020, participants were recruited via web-based diabetes channels and social media. The eligibility requirement was adult age (18 years or older), a good command of the German language, internet access, and a diagnosis of diabetes. All participants gave electronic informed consent before the survey began and were told about the length of time of the survey, which took approximately 15 minutes to complete. Participation was anonymous and voluntary, and the participants could withdraw from it at any time without harm. No financial compensation was offered, and no personal information was collected or stored. Of the 340 participants, 261 (76.8%) completed the survey, thereby forming the total sample of 261 participants. Three participants who stated not to have diabetes were excluded; hence, the final sample comprised 258 participants (192/258, 74.4% female and 66/258, 26.6% male) with complete cases. Multiple entries from the same individual were prevented by using cookies. The study was conducted in accordance with the Declaration of Helsinki and approved by the ethics committee of the medical faculty of the University Duisburg-Essen (19-89-47-BO).

### Measures

#### Overview

The web-based survey contained items on sociodemographic, medical, and mental health data. In addition, we used the validated and well-established UTAUT questionnaire, which assesses health-related internet use, acceptance of eHealth interventions, and barriers and resources of eHealth use [54], and modified it to our research questions based on previous adaptations (Textbox 1). To assess mental health, validated measures were used, such as the Patient Health Questionnaire-2 (PHQ-2) [57], the Generalized Anxiety Disorder Scale-7 (GAD-7) [58], and the Distress Thermometer (DT) [59].

**Adapted items of the Unified Theory of Acceptance and Use of Technology model and references of original studies. Italicized verbalizations have been adapted.**

**Behavioral Intention (=acceptance)**

- “I would like to try a *psychological web-based intervention*.” [52,60,61]
- “I would use a *psychological web-based intervention* if offered to me.” [52,60,61]
- “I would recommend a *psychological web-based intervention* to *my* friend*s*.” [48]

**Social Influence**

- “People close to me would approve *the use of* a *psychological web-based intervention*.” [52,54,60,61]
- “My general practitioner would approve the *application* of a *psychological web-based intervention*.” [52,60,61]
- “My friends would approve a *psychological web-based intervention*.” [28]

**Performance Expectancy**

- “A *psychological web-based intervention* could improve my *general* well-being.” [52,60,61]
- “A *psychological web-based intervention* could help me with *distress*.” [52,60,61]
- “A *psychological web-based intervention* could help me improve my personal *(psychological)* health.” [52,60,61]

**Effort Expectancy**

- “The use of a psychological web-based intervention would not be an additional burden to me.” (Self-constructed)
- “A *psychological web-based intervention* would be easy to operate and comprehend.” [49,52,54,60,61]
- “I could arrange using a *psychological web-based intervention* in my everyday life.” [28]

#### Sociodemographic and Medical Data

Sociodemographic and medical data were assessed using items on gender, age, marital status, having children (aged<18 years), educational level, occupational status, and community size. Age was measured in six categories (18-24, 25-34, 35-44, 45-54, 55-64, and 65 or above). Regarding their medical condition, participants were asked whether they had a mental disorder, diabetes type, how well their diabetes was controlled, how their diabetes was treated, and since when they knew of their diabetes diagnosis. In terms of diabetes treatment, participants could state whether their diabetes was treated with oral medication, insulin, other treatment methods, or not treated, and multiple answers were possible.

#### Acceptance and UTAUT Predictors

Acceptance was operationalized as behavioral intention in accordance with the UTAUT and was measured using three items (Textbox 1). In terms of its specific content, acceptance was defined as the acceptance of a general psychological web-based intervention, which had neither been specified and tailored to patients with diabetes nor offered during this study or later. The underlying UTAUT predictors *PE, EE,* and *SI* were measured using three items each (Textbox 1). Answers were indicated on a 5-point Likert scale ranging from 1 (totally disagree) to 5 (totally agree). As *FC* are based on the UTAUT just as a predictor of use (*use behavior*) and not acceptance itself [54], *FC* have not been included in the statistical analyses. Cronbach α values in this study were .87 for acceptance, .96 for *PE*, .81 for *EE* and .87 for *SI*, proving a high internal consistency.

#### GAD-7 Anxiety

The GAD-7 comprises seven items measuring the frequency of anxiety symptoms over the past 2 weeks on a 4-point Likert scale (0=never to 3=nearly every day). According to previous validation samples, a sum score of ≥5, ≥10, and ≥15 points to mild, moderate, and severe generalized anxiety symptoms, respectively. This assessment instrument has demonstrated high reliability and validity in health care and research [58]. Cronbach α in this study was .91, indicating a high internal consistency.

#### PHQ-2 Depressive Symptoms

The PHQ-2 comprises two items that screen the frequency of depressive symptoms over the past 2 weeks on a 4-point Likert scale (0=never to 3=nearly every day). A sum score of ≥3 serves as a cutoff for major depressive symptoms [57]. In our study, the internal consistency was sufficient, with Cronbach α=.84.

#### DT Distress

The DT [59] involves one visual analog scale from 0 (no distress) to 10 (extreme distress) experienced in the past week. A score of ≥4 indicated increased psychological distress [62].

The items were not randomized or alternated among individuals. The participants were able to change and review their responses while answering. Only completely answered questionnaires could be sent off and were analyzed.

### Statistical Analyses

#### Overview

Data analysis was performed using SPSS 26 (IBM), the macro process by Andrew F Hayes (version 3.3) [63] and the software R [64] (version 3.6.3; R Foundation for Statistical Computing). The level of significance was set at α=.05 (two-sided tests) except for two-tailed *t* tests and analysis of variances (ANOVAs; see *Research Question 1*). First, the internal consistencies and descriptive statistics were calculated. Second, general acceptance was computed and its distribution was assessed (research question 1). In accordance with previous research [28], acceptance (1-5) was categorized by mean in low (1-2.34), moderate (2.35-3.67), and high (3.68-5) acceptance, and the respective frequencies were calculated.

#### Research Question 1: Acceptance and Its Differences by Sociodemographic and Medical Data

Next, the means of acceptance were compared between groups regarding sociodemographic and medical data using *t* tests and ANOVAs to include variables with multiple categories (research question 1). Bonferroni correction was applied to keep the α error low for multiple pairwise comparisons. To prevent the inflation of the α error caused by multiple *t* tests and ANOVAs, we adjusted the respective α level for each *t* test and each ANOVA. For each *t* test, the level of significance was .017 and .007 for each ANOVA.

The normal distribution of acceptance was examined with the Kolmogorov-Smirnov test, skewness, and kurtosis and graphically via a histogram including a normal distribution curve. All these measures detected violations against a normal distribution. Parametric tests were still performed for two reasons. First, according to the central limit theorem, the sampling distribution of the mean of a variable can be safely assumed to be normal if the variable and its mean are normally distributed in the population and the sample size is sufficiently large. We consider our sample size of 258 to be sufficient, because some literature suggests that such an effect already emerges with a sample size of n=30 [65]. In addition, other researchers found acceptance distributions that did not seem to differ from the normal distribution [48], thereby indicating that variable acceptance might be normally distributed in the population. Second, *t* tests and ANOVAs are considered to be robust against violations assuming normal distribution [66].

#### Research Question 2: Predictors of Acceptance

Using multiple hierarchical regression analyses, the predictive models of acceptance were tested and compared (research question 2). The following predictors were included blockwise: (1) sociodemographic and medical variables, (2) mental health variables, and (3) UTAUT predictors. The categorical variable age was dummy coded before being included in the regression analyses; the category with the highest n (age 45-54, n=70) was used as a reference. In addition, the full model was tested against a restricted model (UTAUT predictors only) (research question 3). No multicollinearity could be detected, because all values of the variance inflation factor were <5. To examine the normality of residuals, the Kolmogorov-Smirnov test was computed (*P=*.20) and q-q plots were visually inspected; both showed no signs of violations against normality. Homoscedasticity was proven based on a scatter plot of the standardized residuals and adjusted predicted values.

#### Research Question 3: UTAUT Versus Full Regression Model and Moderator Analyses

In the final step, bootstrapped moderation analyses (model 1) were performed with acceptance as the dependent variable; UTAUT predictors *PE*, *EE*, and *SI* as the respective independent variable; and age or gender as a potential moderator (research question 3). The number of bootstrap samples was set to 10,000. Independent variables were centered, because the value 0 was not defined on a 5-point Likert scale ranging from 1 (totally disagree) to 5 (totally agree).

## Results

### Sociodemographic and Medical Data

Of the 258 participants, 192 (74.4%) women and 66 (25.6%) men participated in the study. Most participants (n=234, 90.7%) were middle age (25-64 years), whereas the most frequent age category was 45-54 years (70/258, 27.1%). Marital status was classified as either mostly *married* or *in a relationship* (174/258, 67.4%). The majority (200/258, 77.5%) had no children aged <18 years. Our sample was highly educated, because 62.5% (162/258) had higher education entrance qualifications or university education; most were employed (160/258, 62%). The community size was nearly balanced, meaning that none of the four community size categories occurred predominantly. Table S1 in Multimedia Appendix 1 provides a comprehensive summary of sociodemographic information.

A mental disorder was present in 27.5% (71/258) participants. Regarding the type of diabetes, 67.4% (174/258) participants had type 1 diabetes, 28.7% (74/258) patients had type 2 diabetes, and 3.9% (10/258) had other types of diabetes. In total, 49.6% (128/258) participants rated their diabetes control as being *good* and 5.4% (14/258) as *not good*. In terms of medical treatment for diabetes, 76% (196/258) used oral medication, 84.1% (217/258) injected insulin, 3.9% (10/258) had other treatment, and 3.5% (9/258) had no treatment. On average, patients had known of their diabetes disease for approximately 17 years (mean 16.98, SD 13.64).

The GAD-7 (mean 6.12, SD 5.20) measures revealed that 29.1% (75/258) of participants had mild, 16.6% (43/258) had moderate, and 7.8% (20/258) had severe anxiety symptoms (Tables S2-S4 in Multimedia Appendix 1). The analyses of measures of PHQ-2 (mean 1.52, SD 1.75) and DT (mean 4.72, SD 2.90) resulted in 21.7% (56/258) and 65.9% (170/258) participants, respectively, reaching the cutoff values of 3 and 4, respectively (Tables S2-S4 in Multimedia Appendix 1). Further information about GAD-7, PHQ-2, and DT measures stratified by gender, age, and diabetes type are displayed in the Multimedia Appendix 1 (Tables S2-S7).

### Research Question 1: Acceptance and Its Differences by Sociodemographic and Medical Data

The general acceptance of e-mental health interventions was moderate, with a mean of 3.02 (SD 1.14). Its distribution can be roughly estimated as one-third for each category; 34.1% (88/258) participants showed low, 37.2% (96/258) showed moderate, and 28.7% (74/258) showed high acceptance. Acceptance differed significantly between female and male participants (*t*_256_=4.21; *P*<.001), with a higher acceptance in women than in men. Having a psychological illness was also significantly associated with higher acceptance ratings (*t*_256_=−4.47; *P*<.001). No differences in acceptance regarding age groups, marital status, having children aged under 18 years, educational status, occupational status, community size, diabetes type, and diabetes control were observed. Table S1 in Multimedia Appendix 1 illustrates acceptance scores as a function of sociodemographic and medical data.

### Research Question 2: Predictors of Acceptance

The multiple hierarchical regression analysis revealed that the sociodemographic and medical variables included in the first step explained 14.2% of the variance in acceptance (*R*²=0.142; *F*_7,250_=5.903; *P*<.001). Therefore, gender (β=−.20; *P*=.001) and having a mental disorder (β=.24; *P*<.001) significantly predicted acceptance. The mental health variables included in the second step (*R*²=0.204; *F*_10,247_=6.347; *P*<.001) significantly increased the explained variance (Δ*R*²=0.063; *F*_3,247_=22.166; *P*<.001), even though none of these variables were significant predictors of acceptance. However, generalized anxiety (GAD-7) was very close to statistical significance (*P*=.05). The UTAUT predictors included in the last step (*R*²=0.770; *F*_13,244_=62.966; *P*<.001) changed the explained variance significantly by 56.6% (Δ*R*²=0.566; *F*_3,244_=200.451; *P*<.001), further resulting in a total percentage of explained variance of 77%. *PE* (β=.50; *P*<.001), *EE* (β=.15; *P=*.001), and *SI* (β=.28; *P*<.001) significantly predicted acceptance. In addition, the DT was a significant predictor of acceptance in the full regression model (β=.11; *P*=.03). Table 1 presents the regression parameters of the hierarchical regression model of acceptance.

**Table 1:** Hierarchical regression model of acceptance. N=258.^a^

Predictor		Standardized coefficient β	Unstandardized coefficient β, B	T	Determination coefficient, R²		Changes in R², ΔR²		*P* value	
**Step 1: sociodemographic and medical predictors**						0.142		0.142		
	Gender	−.204	−.533	−3.368					.001	
	Age^b^	−.053, .083	−.327, .260	−0.696, 1.193					.42	
	Mental disorder	.240	.612	4.056					<.001	
**Step 2: mental health variables**						0.204		0.063		
	GAD-7^c^	.216	.047	1.934					.05	
	PHQ-2^d^	−.028	−.018	−0.273					.76	
	Distress Thermometer [59]	.119	.047	1.383					.17	
**Step 3: UTAUT^e^ predictors**						0.770		0.566		
	Performance expectancy	.503	.487	10.340					<.001	
	Effort expectancy	.150	.181	3.366					.001	
	Social influence	.282	.328	6.268					<.001	

^a^In Steps 2 and 3, only the newly included variables are presented.

^b^Age was measured in categories and therefore has been included as a dummy variable. The category with the highest n (age: 45-54 years, n=70) was used as a reference. For *β,* B, and T minima, the maxima of each group contrast are presented. The *P* value was aggregated using the statistical software R; no single *P* value of each contrast reached statistical significance.

^c^GAD-7: Generalized Anxiety Disorder Scale-7 [58].

^d^PHQ-2: Patient Health Questionnaire-2 [57].

^e^UTAUT: Unified Theory of Acceptance and Use of Technology.

### Research Question 3: UTAUT Versus Full Regression Model and Moderator Analyses

The comparison between our extended UTAUT model (13 predictors) and the UTAUT-only model (three predictors) revealed no significant difference in explained variance in acceptance (*F*_10,244_=1.567; *P=*.12).

Several moderation analyses have been computed to prove whether age and gender work as moderators, as postulated by the UTAUT [54]. None of them turned out to be statistically significant. Thus, neither gender nor age moderated the relationship between acceptance and the respective UTAUT predictor *PE* (gender: *P*=.61, *F*_1,254_=0.269; age: *P*=.34, *F*_1,254_=0.922), *EE* (gender: *P*=.33, *F*_1,254_=0.962; age: *P*=.28, *F*_1,254_=1.162), or *SI* (gender: *P*=.10, *F*_1,254_=2.726; age: *P*=.53, *F*_1,254_=0.400).

## Discussion

### Principal Findings

This study examined the acceptance of e-mental health interventions among patients with diabetes and explored factors influencing patients’ intention to use such interventions. The overall acceptance of e-mental health interventions was moderate. Acceptance was associated with gender and mental illness, because women and participants with mental illness had a significantly higher acceptance than men and participants without a stated mental disorder. No difference in acceptance regarding other sociodemographic and medical data were observed, whereas diabetes type only failed to show a statistical significance based on an adapted level of significance (see *Statistical Analyses* section). In the full regression model, the acceptance of e-mental health interventions was significantly predicted by mental health variable *distress* (DT) as well as the UTAUT predictors *PE*, *EE*, and *SI*. The UTAUT predictors (restricted model with UTAUT predictors only) reached a similar explained variance (75%) in acceptance as the full regression model (13 predictors). Neither gender nor age were moderators of the relationship between each UTAUT predictor and acceptance, which contradicts one postulation of the UTAUT [54].

### Comparison With Previous Work

Our results confirm the high psychological vulnerability of patients with diabetes, which previous studies had emphasized [5-15], as approximately 21.7% (56/258) and 65.9% (170/258) of our participants expressed indications of major depression (PHQ-2≥3) and elevated distress (DT≥4), respectively. Measures of the GAD-7 showed that 16.7% (43/258; GAD-7≥10) and 7.8% (20/258; GAD-7≥15) had moderate and severe levels of anxiety, respectively.

Despite the proven high psychological vulnerability of patients with diabetes, for example, the increased risk of developing depressive symptoms caused by, for example, heavy demands and major distress or medical complications and constraints this disease and its treatment poses [67,68], research on determining the acceptance of e-mental health interventions, which could have provided help flexibly, and its underlying barriers and resources have been scarce. In particular, research using validated measures (eg, UTAUT) assessing the acceptance of e-mental health interventions in people with diabetes has been lacking. To our knowledge, only one study took on this major research subject [52] and conducted important research groundwork by doing so. Nevertheless, our study was imperative and is of great significance for the following reasons. First, their focus was on determining acceptance and examining the effectiveness of an offered acceptance-facilitating intervention (AFI) and not on assessing acceptance and its predictors and testing the viability of the UTAUT model. Second, the sample size of patients with diabetes was smaller, and the study was conducted 7 years ago. Third, their measurement methods implied noteworthy deficiencies; although relying on the UTAUT, *FC* were regarded as a predictor of acceptance, and not just a predictor of use [54]. In addition, behavioral intention as an operational construct of acceptance is measured using four items instead of three, which is also discordant to the UTAUT [54]. In particular, by deviating in this matter and adding a fourth self-generated item covering willingness to pay for e-mental health interventions, the authors diverge from previous research and respective UTAUT adaptations [28,47,49], not taking advantage of the viability and validity of the UTAUT and its adaptations to the field of e-(mental) health acceptance.

As stated above, the research group investigated patients with diabetes’ behavioral intention to use internet-based interventions for depression [52]. The measured acceptance ratings differed from our results (mean 3.02, SD 1.14). Transforming their acceptance scale (4-20) to our acceptance scale (1-5), the average acceptance was lower than that observed in our diabetes sample (control group: mean 2.42, SD 1.07; intervention group: mean 2.64, SD 1.18), but the variance was comparable. Different selection methods may have contributed to these differences. In contrast to the recruitment in the aforementioned study, recruitment was conducted via the internet, resulting in a sample of patients with diabetes that may have been more open to and interested in e-mental health interventions. On the other hand, the passage of time and increasing digitalization and experiences with and knowledge of e(-mental) health tools may also have accounted for these differences. In addition, by adding a fourth finance-based self-generated item of behavioral intention, acceptance ratings might not be comparable. Contrary to the expectations of the researchers, their AFI had no significant effect on acceptance and its predictors. However, subgroup analyses revealed a significant effect for female participants and yielded a trend for younger (<59), depressed, diabetes-related distressed participants, and for those with a low frequency of internet usage to benefit from their AFI [52]. Therefore, future AFIs should probably be tailored to the specific needs of the respective subpopulations.

Studies assessing the acceptance of e-mental health interventions in general found the following variables to be significantly associated with acceptance: gender [55], age [55], education [55], anxiety [48], internet anxiety [48], prior e-mental health use [55,69], and current or past mental disorders [55]. An investigation of acceptance of a web-based aftercare in a mixed inpatient sample with subgroups from different medical clinics revealed that acceptance was significantly associated with (younger) age, (higher) education, stress due to permanent availability, private internet access, and prior eHealth use [28]. With regard to a diabetes-specific web-based platform for patients with type 2 diabetes, stating interest was significantly associated with being male, younger age, higher education, and shorter duration of type 2 diabetes [70].

In line with previous research, acceptance ratings in this study were significantly associated with gender and current or past mental illness. However, contrary to previous studies [25,52], acceptance ratings in this study were significantly higher for women than for men. Age and educational background were not significantly associated with acceptance. Nevertheless, acceptance in this study showed a descriptive trend toward a higher acceptance in younger participants.

Exploring the underlying predictors of eHealth acceptance, previous research adapted the UTAUT and identified the following significant predictors: the UTAUT predictors *PE* [28,50,51,71], *EE* [28,50,51,71] and *SI* [28,50,51] and other predictors namely *perceived reliability* [51], *stress due to permanent availability* [28], *perceived security* [71], *technology anxiety* [50], and *resistance to change* [50]. *FC* are the fourth core predictors of UTAUT, which is supposed to significantly predict the actual usage, but not the behavioral intention (=acceptance) [54]. However, some studies incorporated *FC* as a fourth UTAUT predictor of acceptance [28,50,51,71], generating inconsistent results. In 2 studies, *FC* was a significant predictor of acceptance, whereas others were not. In case of significance, its predictive value can be regarded as rather low, for example, reaching correlations of *r*=0.12 with acceptance [71].

Our results support the viability of UTAUT in determining e-mental health acceptance. The proportion of explained variance by *PE*, *EE*, and *SI* was high in this study (75%) and comparable with those of the original UTAUT validation study (70%) [54]. Confirming previous research, in this study *PE* was the key predictor of acceptance [28,49,50,54,72].

Acceptance operationalized as *behavioral intention* can be regarded as a significant and highly valid predictor of actual usage behavior. According to the UTAUT, *behavioral intention* is a significant predictor of actual use, and both correlate with *r*=0.59 [54]. In addition, prior eHealth research emphasized the major role of acceptance regarding a valid prediction of the actual eHealth usage: predicting the usage of mobile health services, *behavioral intention* had a significant effect and reached notable standardized regression coefficients of β*=*.415 [50] and β*=*.372 [51] in the respective structural model. Moreover, acceptance can predict the future use of eHealth interventions, as higher ratings of acceptance led to higher use of an unguided web-based intervention for chronic pain [48]. Nevertheless, the majority of studies on e(-mental) health acceptance do not measure its actual use (eg, uptake rate of an offered intervention), and if so, usage behavior was assessed retrospectively. To prove the prospective validity of acceptance, future research should include actual use and uptake rates as an outcome measure and assess acceptance in advance.

Comparing the acceptance and use of eHealth in terms of significant associations with sociodemographic variables, the findings appear to be similar. For both age and education, significant associations are generated, as younger age and higher educational level are associated with higher acceptance and higher use [28,55,70,73-75]. In contrast to acceptance, eHealth use was found to be associated with income and living conditions, finding that not living alone and higher income were associated with higher usage behavior [73,74]. Gender with regard to eHealth use has resulted in inconsistent results. A recent review found 8 studies with no significant association between gender and eHealth use and 5 studies stating a significant association between gender and eHealth use in patients with chronic diseases [73]. Increasing the heterogeneity of these results, 3 of the 5 studies found that being female is associated with an increased usage behavior, whereas 2 studies revealed that males showed a higher use of eHealth services.

These heterogeneous findings are of central importance, as they are a valid reminder of the limitations of research on eHealth acceptance and eHealth use and the variety of (unknown) underlying factors. In our understanding, the following factors may influence the acceptance and use of eHealth and should be regarded in terms of interpreting research results: measurement method (eg, UTAUT or not), recruitment method (web-based or not), sociodemographic characteristics (gender, age, education, income, and living conditions), duration of illness, user type (inpatient, outpatient, staff, and public), technical type of delivery (app, mobile, tablet, and laptop), guidance (unguided vs guided), target population (healthy, type of illness, and chronic disease), type of eHealth service (intervention program, education, communication, and technical devices), and the specificity of the eHealth service (general health, general mental health, and specific for current disease).

### Limitations

The following limitations should be considered while interpreting the results. First, because our study was a web-based survey, patients were required to have internet access. As the probability of internet access declines with age [76], we received a younger diabetes sample than that present in the general population. Consequently, we had more patients with type 1 diabetes (174/258, 67.4%) than patients with type 2 diabetes (74/258, 28.7%), because type 2 is an acquired form of diabetes in older age. Therefore, our sample does not reflect the typical distribution of 90% of patients with type 2 diabetes [77]. The composition of the investigated sample was mostly female (approximately 192/258, 75%). Owing to this selection bias, the generalizability of our results may be reduced.

Second, these compositional specifications may not only limit generalizability, but also bias acceptance ratings, because participants recruited via the internet may be more open to and interested in e-mental health interventions. In addition, female patients had significantly higher acceptance ratings than male patients. Furthermore, in a descriptive view, younger patients had higher acceptance ratings than older patients. In addition, acceptance may be overestimated, because patients with type 1 diabetes showed a higher acceptance than patients with type 2 diabetes, but only in a descriptive view based on our adapted level of significance of 0.007 (see *Statistical Analyses*).

Third, all data were self-reported. This method is prone to the *common method* bias because of the shared method variance [78]. Counteracting this limitation, the instruments used had a sufficient internal reliability, the survey had a short length, and the patients were well-educated because these points are known to mitigate the *common method* bias.

Fourth, we measured acceptance as the *behavioral intention*, which corresponds to previous studies [28,48,49,51,54,79-81]. However, direct conclusions from the intention to use an e-mental health intervention to its actual usage cannot be drawn because of the intention-behavior gap [82]. Thus, acceptance can be assessed as a predictive function of behavioral intention, and previous studies have proven its validity [48,50,51,54].

Fifth, our study design was based on assessing mental health only through symptom-based measures, which were oriented on diagnostic criteria. Although we used established and validated measures, which is one strength of our study (PHQ-2, DT, and GAD-7), it could also be beneficial to include the mental health–related quality of life measures.

Sixth, the cross-sectional study design is not suited to identify and account for factors and predictors of acceptance and use of eHealth interventions that may interact or change over time and depend on technological progress. In addition, because of the cross-sectional design, no statements can be made regarding causality.

To overcome these limitations, future research should add the uptake rate as an outcome measure and focus on assessing acceptance and its barriers and resources in a longitudinal design with different target groups and a balanced composition of gender and age. Furthermore, future research should add health-related quality of life measures to investigate how predictive these measures can be regarding e-mental health acceptance and if those might be even more predictive than symptom-based validated measures of mental health. Moreover, qualitative or mixed methods research could help identify the unknown predictors of e-mental health acceptance and therefore add richness to the understanding of underlying barriers and resources of e-mental health acceptance in general, particularly for e-mental health interventions in patients with diabetes.

### Conclusions

This study supports the viability of the UTAUT model and its predictors in assessing the acceptance of e-mental health interventions in patients with diabetes. The UTAUT model, comprising the predictors *of PE*, *EE*, and *SI*, reached a notable amount of explained variance in the acceptance of 75%. This UTAUT model was compared with a full model including 13 predictors including sociodemographic, medical, and mental health variables and explained a similar variance in acceptance.

To conclude, the UTAUT model (here, 9 items) turned out to be a very useful and efficient method for measuring the e-mental health acceptance of patients with diabetes. Due to the close link between acceptance and use, AFIs should be fostered to bring forward the establishment of effective e-mental health interventions in psychodiabetology. These AFIs should focus on *PE*, *EE*, and *SI* to increase the acceptance of easily accessible and location-flexible e-mental health interventions, which are even more vitally needed these days given the ongoing pandemic and in light of the high (psychological) vulnerability of patients with diabetes.

However, future research should also include the actual use behavior as an outcome measure to test the (prospective) validity of acceptance predictions for the actual use of e-mental health interventions.

## Appendix

Multimedia Appendix 1 Statistical data tables of differences in acceptance and prevalence of anxiety, depressive symptoms, and distress.

## Abbreviations

AFI: acceptance-facilitating intervention
ANOVA: analysis of variance
DT: Distress Thermometer
EE: effort expectancy
FC: facilitating conditions
GAD-7: Generalized Anxiety Disorder Scale-7
PE: performance expectancy
PHQ-2: Patient Health Questionnaire-2
SI: social influence
UTAUT: Unified Theory of Acceptance and Use of Technology
