# Supplementary material for: Determining Acceptance of e-Mental Health Interventions in Digital Psychodiabetology Using a Quantitative Web-Based Survey: Cross-sectional Study
Source: JMIR Form Res. 2021 Jul 30;5(7):e27436. doi: 10.2196/27436 (PMC8367156; doi:10.2196/27436)
Supplement: Multimedia Appendix 1 [file formative_v5i7e27436_app1.doc]

**Determining Acceptance of e-Mental Health Interventions in Digital Psychodiabetology Using a Quantitative Web-Based Survey: Cross-sectional Study**

Mirjam Damerau^1†^, Martin Teufel^1^, Venja Musche^1^, Hannah Dinse^1^, Adam Schweda^1^, Jil Beckord^1^, Jasmin Steinbach^1^, Kira Schmidt^1^, Eva-Maria Skoda^1^, Alexander Bäuerle^1^

^1^Clinic for Psychosomatic Medicine and Psychotherapy, LVR-University Hospital Essen, University of Duisburg Essen, Germany.

^†^Corresponding author: Mirjam Damerau, phone: +49(201)438755228, fax: +49(201)438755255

E-Mail: Mirjam.damerau@uni-due.de

## Multimedia Appendix 1

This is a Multimedia Appendix to a full manuscript published in the J Med Internet Res. For full copyright and citation information see http://dx.doi.org/10.2196/jmir.27436

**Table S1.** Differences in acceptance by socio-demographic and medical data (N = 258).

| Variable | |  | n (%) | | | Mean (SD) | | | | | | | Test | | | | | *P* value | |
| --- | --- | --- | --- | --- | --- | --- | --- | --- | --- | --- | --- | --- | --- | --- | --- | --- | --- | --- | --- |
| **Sex** | | | | | | | | | | | | | |  |  |  |  | |  |
|  | Female | | 192 (74.4) | | | 3.19 (1.10) | | | | | | | *t*_256_=4.21 | | | | | <.001^a^ | |
|  | Male | | 66 (25.6) | | | 2.53 (1.11) | | | | | | |  | | | | |  | |
| **Age in years** | |  |  | | |  | | | | | | |  | | | | |  | |
|  | 18-24 | | 15 (5.8) | | | 3.47 (.95) | | | | | | | *F*_5,252_=1.99 | | | | | .08^b^ | |
|  | 25-34 | | 48 (18.6) | | | 3.32 (1.01) | | | | | | |  | | | | |  | |
|  | 35-44 | | 65 (25.2) | | | 2.99 (1.16) | | | | | | |  | | | | |  | |
|  | 45-54 | | 70 (27.1) | | | 2.86 (1.16) | | | | | | |  | | | | |  | |
|  | 55-64 | | 51 (19.8) | | | 2.97 (1.19) | | | | | | |  | | | | |  | |
|  | 65 or above | | 9 (3.5) | | | 2.41 (1.16) | | | | | | |  | | | | |  | |
| **Marital status** | |  |  | | |  | | | | | | |  | | | | |  | |
|  | Single | | 65 (25.2) | | | 3.15 (1.18) | | | | | | | *F*_5,252_=1.05 | | | | | .39^b^ | |
|  | Married | | 119 (46.1) | | | 2.95 (1.63) | | | | | | |  | | | | |  | |
|  | In a relationship | | 55 (21.3) | | | 3.04 (1.02) | | | | | | |  | | | | |  | |
|  | Divorced/separated | | 14 (5.4) | | | 3.19 (1.22) | | | | | | |  | | | | |  | |
|  | Widowed | | 3 (1.2) | | | 2.78 (0.96) | | | | | | |  | | | | |  | |
|  | Other | | 2 (0.8) | | | 1.50 (1.14) | | | | | | |  | | | | |  | |
| **Children <18** | |  |  | | |  | | | | | | |  | | | | |  | |
|  | No | | 200 (77.5) | | | 3.01 (1.12) | | | | | | | t_256_=.158 | | | | | .86^a^ | |
|  | Yes | | 58 (22.5) | | | 3.04 (1.21) | | | | | | |  | | | | |  | |
| **Educational level** | | | |  |  |  | | | | | | |  | | | | |  | |
|  | University education | | 75 (29.1) | | | 3.10 (1.11) | | | | | | | *F*_5,252_=.89 | | | | | .49^b^ | |
|  | Higher education entrance qualification | | 87 (33.7) | | | 3.09 (1.10) | | | | | | |  | | | | |  | |
|  | Secondary education | | 57 (22.1) | | | 2.88 (1.14) | | | | | | |  | | | | |  | |
|  | Lower secondary education | | 29 (11.2) | | | 2.74 (1.27) | | | | | | |  | | | | |  | |
|  | No qualification | | 2 (0.8) | | | 3.67 (1.41) | | | | | | |  | | | | |  | |
|  | Other | | 8 (3.1) | | | 3.29 (1.28) | | | | | | |  | | | | |  | |
| **Occupational status** | | | | | | | | | |  |  |  |  | | | | |  | |
|  | Trainee/student | | 15 (5.8) | | | 3.38 (.99) | | | | | | | *F*_7,250_=.51 | | | | | .83^b^ | |
|  | Unemployed | | 14 (5.4) | | | 3.02 (.72) | | | | | | |  | | | | |  | |
|  | Partly employed | | 54 (20.9) | | | 3.06 (1.17) | | | | | | |  | | | | |  | |
|  | Full employed | | 106 (41.1) | | | 2.90 (1.08) | | | | | | |  | | | | |  | |
|  | Unfit for work | | 4 (1.6) | | | 2.92 (1.55) | | | | | | |  | | | | |  | |
|  | Sick leave | | 14 (5.4) | | | 3.24 (1.55) | | | | | | |  | | | | |  | |
|  | Retired | | 31 (12.0) | | | 3.00 (1.27) | | | | | | |  | | | | |  | |
|  | Other | | 20 (7.8) | | | 3.17 (1.16) | | | | | | |  | | | | |  | |
| **Community size** | | | | | | |  |  |  | | | |  | | | | |  | |
|  | ≥ 100,000 residents^c^ | | 85 (32.9) | | | 3.19 (1.12) | | | | | | | *F*_3,254_=1.02 | | | | | .38^b^ | |
|  | ≥ 20,000 residents^d^ | | 74 (28.7) | | | 2.88 (1.11) | | | | | | |  | | | | |  | |
|  | ≥ 5,000 residents^e^ | | 42 (16.3) | | | 2.98 (1.18) | | | | | | |  | | | | |  | |
|  | < 5,000 residents^f^ | | 57 (22.1) | | | 2.98 (1.18) | | | | | | |  | | | | |  | |
| **Mental disorder(s)** | | | | | | | | | |  |  |  |  | | | | |  | |
|  | No | | 187 (72.5) | | | 2.83 (1.12) | | | | | | | *t*_256_=-4.47 | | | | | <.001^a^ | |
|  | Yes | | 71 (27.5) | | | 3.52 (1.04) | | | | | | |  | | | | |  | |
| **Diabetes type** | | | | | | |  |  |  | | | |  | | | | |  | |
|  | Type 1 | | 174 (67.4) | | | 3.11 (1.09) | | | | | | | *F*_2,255_=3.40 | | | | | .04^b^ | |
|  | Type 2 | | 74 (28.7) | | | 2.74 (1.12) | | | | | | |  | | | | |  | |
|  | Other type of diabetes | | 10 (3.9) | | | 3.40 (1.30) | | | | | | |  | | | | |  | |
| **Diabetes control** | | | | | | |  |  |  | | | |  | | | | |  | |
|  | good | | 128 (49.6) | | | 2.89 (1.16) | | | | | | | *F*_3,254_=1.89 | | | | | .13^b^ | |
|  | average | | 110 (42.6) | | | 3.18 (1.10) | | | | | | |  | | | | |  | |
|  | not good | | 14 (5.4) | | | 3.24 (0.97) | | | | | | |  | | | | |  | |
|  | could not rate | | 6 (2.3) | | | 2.50 (1.46) | | | | | | |  | | | | |  | |

Note. In Germany, community size is measured using four categories with ^c^ = metropolis, ^d^ = medium-sized town, ^e^ = small town and ^f^ = rural town.

**^a^** significant if *P*<.017

^b^ significant if *P*<.007

**Table S2.** Prevalence of GAD-7, PHQ-2 and DT for sex.

|  | | **Total** | **Sex** | |
| --- | --- | --- | --- | --- |
|  | | (N = 258) | Female  (N = 192) | Male  (N = 66) |
|  | | n (%) | n (%) | n (%) |
| **GAD-7^a^** | |  |  |  |
|  | < 5 | 120 (46.5) | 76 (39.6) | 44 (66.7) |
|  | ≥ 5 | 75 (29.1) | 60 (31.3) | 15 (22.7) |
|  | ≥ 10 | 43 (16.6) | 38 (19.8) | 5 (7.6) |
|  | ≥ 15 | 20 (7.8) | 18 (9.3) | 2 (3.0) |
| **PHQ-2^b^** | |  |  |  |
|  | < 3 | 202 (78.3) | 144 (75.0) | 58 (87.9) |
|  | ≥ 3 | 56 (21.7) | 48 (25.0) | 8 (12.1) |
| **DT^c^** | |  |  |  |
|  | < 4 | 88 (34.1) | 53 (27.6) | 35 (53.0) |
| ≥ 4 | | 170 (65.9) | 139 (72.4) | 31 (47.0) |

Note. ^a^Generalized Anxiety Disorder-Scale-7 (GAD-7), ^b^Patient Health Questionnaire-2 (PHQ-2), ^c^Distress Thermometer.

**Table S3.** Prevalence of GAD-7, PHQ-2 and DT for age.

|  |  | | **Total** | **Age – categories in years** | | | | | |
| --- | --- | --- | --- | --- | --- | --- | --- | --- | --- |
|  |  | | (N = 258) | 18-24  (N = 15) | 25-34  (N = 48) | 35-44  (N = 65) | 45-54  (N = 70) | 55-64  (N = 51) | 65-74  (N = 9) |
|  |  | | n (%) | n (%) | n (%) | n (%) | n (%) | n (%) | n (%) |
|  | **GAD-7^a^** | |  |  |  |  |  |  |  |
|  | | < 5 | 120 (46.5) | 4 (26.7) | 21 (43.8) | 28 (43.1) | 33 (47.1) | 27 (52.9) | 7 (77.8) |
|  | | ≥ 5 | 75 (29.1) | 4 (26.6) | 14 (29.1) | 21 (32.3) | 21 (30.0) | 14 (27.5) | 1 (11.1) |
|  | | ≥ 10 | 43 (16.6) | 3 (20.0) | 10 (20.9) | 12 (18.4) | 11 (15.8) | 7 (13.7) | 0 (0.0) |
|  | | ≥ 15 | 20 (7.8) | 4 (26.7) | 3 (6.2) | 4 (6.2) | 5 (7.1) | 3 (5.9) | 1 (11.1) |
|  | **PHQ-2^b^** | |  |  |  |  |  |  |  |
|  | | < 3 | 202 (78.3) | 10 (66.7) | 42 (87.5) | 51 (78.5) | 19 (27.1) | 38 (74.5) | 8 (88.9) |
|  | | ≥ 3 | 56 (21.7) | 5 (33.3) | 6 (12.5) | 14 (21.5) | 51 (72.9) | 13 (25.5) | 1 (11.1) |
|  | **DT^c^** | |  |  |  |  |  |  |  |
|  | | < 4 | 88 (34.1) | 3 (20.0) | 15 (31.3) | 18 (27.7) | 24 (34.3) | 20 (39.2) | 8 (88.9) |
| ≥ 4 | | | 170 (65.9) | 12 (80.0) | 33 (68.7) | 47 (72.3) | 46 (65.7) | 31 (60.8) | 1 (11.1) |

Note. ^a^Generalized Anxiety Disorder-Scale-7 (GAD-7), ^b^Patient Health Questionnaire-2 (PHQ-2), ^c^Distress Thermometer.

**Table S4.** Prevalence of GAD-7, PHQ-2 and DT for Diabetes type.

|  |  | | **Total** | **Diabetes Type** | |
| --- | --- | --- | --- | --- | --- |
|  |  | | (N = 258) | Type 1  (N = 174) | Type 2  (N = 74) |
|  |  | | n (%) | n (%) | n (%) |
|  | **GAD-7^a^** | |  |  |  |
|  | | < 5 | 120 (46.5) | 80 (46.0) | 34 (40.5) |
|  | | ≥ 5 | 75 (29.1) | 53 (30.4) | 20 (32.5) |
|  | | ≥ 10 | 43 (16.6) | 31 (17.9) | 11 (14.8) |
|  | | ≥ 15 | 20 (7.8) | 10 (5.7) | 9 (12.2) |
|  | **PHQ-2^b^** | |  |  |  |
|  | | < 3 | 202 (78.3) | 139 (79.9) | 55 (74.3) |
|  | | ≥ 3 | 56 (21.7) | 35 (20.1) | 19 (25.7) |
|  | **DT^c^** | |  |  |  |
|  | | < 4 | 88 (34.1) | 54 (31.0) | 29 (39.2) |
| ≥ 4 | | | 170 (65.9) | 120 (69.0) | 45 (60.8) |

Note. ^a^Generalized Anxiety Disorder-Scale-7 (GAD-7), ^b^Patient Health Questionnaire-2 (PHQ-2), ^c^Distress Thermometer.

**Table S5.** Means (standard deviation) of generalized anxiety symptoms,

depression symptoms and psychological distress, stratified by gender.

|  | **Total** | **Sex** | |  |
| --- | --- | --- | --- | --- |
|  | (N = 258) | Female  (N = 192) | Male  (N = 66) | |
|  | Mean (SD) | Mean (SD) | Mean (SD) | |
| **GAD-7^a^** | 6.12 (5.20) | 6.85 (5.19) | 4.00 (4.66) | |
| **PHQ-2^b^** | 1.52 (1.75) | 1.68 (1.78) | 1.03 (1.57) | |
| **DT^c^** | 4.72 (2.90) | 5.16 (2.80) | 3.44 (2.80) | |

Note. ^a^Generalized Anxiety Disorder-Scale-7 (GAD-7), ^b^Patient Health Questionnaire-2 (PHQ-2), ^c^Distress Thermometer.

**Table S6.** Means of GAD-7 sum scores, PHQ-2 sum scores, and DT, stratified by age.

|  | **Total** | **Age – categories in years** | | | | | |  |
| --- | --- | --- | --- | --- | --- | --- | --- | --- |
|  | (N = 258) | 18-24  (N = 15) | 25-34  (N = 48) | 35-44  (N = 65) | 45-54  (N = 70) | 55-64  (N = 51) | 65-74  (N = 9) | |
|  | Mean (SD) | Mean (SD) | Mean (SD) | Mean (SD) | Mean (SD) | Mean (SD) | Mean (SD) | |
| **GAD-7^a^** | 6.12 (5.20) | 9.93 (6.53) | 6.33 (4.71) | 6.20 (4.67) | 5.99 (5.51) | 5.31 (5.13) | 3.67 (5.10) | |
| **PHQ-2^b^** | 1.52 (1.75) | 2.27 (2.25) | 1.25 (1.56) | 1.51 (1.63) | 1.61 (1.84) | 1.59 (1.81) | 0.56 (1.33) | |
| **DT^c^** | 4.72 (2.90) | 5.33 (2.72) | 4.92 (2.61) | 5.00 (2.84) | 4.73 (2.97) | 4.43 (2.99) | 2.11 (3.14) | |

Note. ^a^Generalized Anxiety Disorder-Scale-7 (GAD-7), ^b^Patient Health Questionnaire-2 (PHQ-2), ^c^Distress Thermometer.

**Table S7.** Means of GAD-7 sum scores, PHQ-2 sum scores

and DT stratified by diabetes type.

|  | **Total** | **Diabetes Type** | |  |
| --- | --- | --- | --- | --- |
|  | (N = 258) | Type 1  (N = 174) | Type 2  (N = 74) | |
|  | Mean (SD) | Mean (SD) | Mean (SD) | |
| **GAD-7^a^** | 6.12 (5.20) | 6.09 (4.90) | 6.39 (5.85) | |
| **PHQ-2^b^** | 1.52 (1.75) | 1.48 (1.64) | 1.61 (1.94) | |
| **DT^c^** | 4.72 (2.90) | 4.77 (2.72) | 4.69 (3.30) | |

Note. ^a^Generalized Anxiety Disorder-Scale-7 (GAD-7), ^b^Patient Health Questionnaire-2 (PHQ-2), ^c^Distress Thermometer.
